# An integrative analysis reveals the mechanism of plastic stabilizers inducing breast cancer

**DOI:** 10.1371/journal.pcbi.1014025

**Published:** 2026-03-06

**Authors:** Xingfa Huo, Xueqin Duan, Xiaojuan Huang, Linyuan Xue, Lantao Zhao, Yufeng Li, Xiaochun Zhang, Na Zhou

**Affiliations:** 1 Precision Medicine Center of Oncology, The Affiliated Hospital of Qingdao University, Qingdao, China; 2 Institute of Cancer Research, Qingdao University, Qingdao, China; 3 Department of Anesthesiology, the Affiliated Hospital of Qingdao University, Qingdao, China; University Hospital Schleswig-Holstein - Campus Kiel: Universitatsklinikum Schleswig-Holstein, GERMANY

## Abstract

Plastic stabilizers (PSs) are chemical additives that are widely used to inhibit the degradation of plastics. However, their safety concerns and potential carcinogenic risks remain unclear. This study employed network toxicology strategies to elucidate the potential toxic effects and underlying molecular mechanisms of representative PSs, including 2,6-di-tert-butylphenol (2,6-DTB), tert-butylhydroquinone (TBHQ), and 2-(2H-benzotriazol-2-yl)-4,6-di-tert-pentylphenol (UV-328) in breast cancer (BC). Herein, we identified 69 potential genes related to PSs exposure and BC, and optimized five core targets: *GSK3B*, *MAPK14*, *PARP1*, *PIM1*, and *TRDMT1*, through subsequent LASSO and SVM algorithms. Based on these core genes, we constructed risk score and nomogram models, both of which revealed that high expression of these five core genes predicts poor prognosis in BC patients. Additionally, molecular docking and dynamic simulations indicated high-affinity interactions between PSs and these core targets (binding energies < -5 kcal/mol). Further correlation analysis with prediction analysis of microarray 50 (PAM50) revealed increased expression of all core genes in the basal-like subtype, especially PIM1 and TRDMT1, which also exhibited the highest risk scores. *In vitro*, PSs transcriptionally upregulated *MAPK14*, *PIM1*, and *TRDMT1*, with *STAT3* mediating their transcription. Importantly, cell counting kit-8 and wound healing assays demonstrated that PSs promote BC cell proliferation and migration. Our research re-evaluates the carcinogenic risks of plastic stabilizers and suggests that PSs may enhance breast cancer progression via targets such as *MAPK14*, *PIM1*, and *TRDMT1*. This study introduces a new approach for evaluating the safety of plastic additives and offers novel insights into the toxicological effects of PSs.

## Author summary

Plastic stabilizers are ubiquitous chemical additives used to prevent plastic degradation, yet their potential role in cancer development has been largely overlooked. This study integrates computational network toxicology with biological validation to examine the carcinogenic risks of three common stabilizers: 2,6-DTB, TBHQ, and UV-328. We discovered that these chemicals specifically target and activate core genes—including MAPK14, PIM1, and TRDMT1—associated with breast cancer. Our experiments demonstrate that these stabilizers bind directly to these molecular targets, thereby driving cancer cell proliferation and migration. Furthermore, clinical analysis indicates that elevated levels of these genes correlate with poorer survival outcomes in breast cancer patients. These findings provide evidence that plastic stabilizers are not merely inert additives but potential environmental drivers of tumor progression. By elucidating the molecular mechanism linking plastic additives to cancer aggression, this research offers a scientific basis for re-evaluating the safety of these chemicals and underscores the urgent need for stricter risk assessment of plastic-associated compounds.

## 1. Introduction

Plastic stabilizers (PSs) are a class of chemical additives extensively utilized to prevent plastic degradation and aging for a longer service life. Among them, 2,6-DI-Tert-butylphenol (2,6-DTB), tert-butylhydroquinone (TBHQ), and 2-(2H-benzotriazol-2-yl)-4,6-di-tert-pentylphenol (UV-328) are the most prevalent PSs [1,2]. Their high stability and strong lipophilicity led to persistence and bioaccumulation in humans and the environment [3]. Such characteristics of PSs induced potential endocrine disruption and long-term health risks have attracted growing concern, while precise molecular mechanisms underlying their toxicity and biological effects remain unclear. However, conventional toxicological testing often focuses on single molecular targets, failing to capture complex biological interactions. This underscores the urgent need for comprehensive assessment strategies to evaluate the health risks of PSs.

Breast cancer (BC) as one of the most predominant malignant tumors among women has complex etiological factors involving genetic susceptibility, endocrine regulation and behavioral factors. In recent years, the role of environmental pollutants in breast cancer has attracted increasing attention. Multiple studies have indicated that long-term exposure to air pollutants such as PM2.5, PM10, and NO₂ significantly increased risk of breast cancer [4–6]. In addition, polycyclic aromatic hydrocarbons (PAHs), polychlorinated biphenyls (PCBs), and benzo[a]pyrene (BaP) may disrupt endocrine, activate estrogen receptor pathways, or cause DNA damage, leading to promotion of breast cancer [7,8]. Although both plastic stabilizers and air pollutants are widely prevalent environmental contaminants, the potential role of chronic exposure to plastic stabilizers in breast carcinogenesis and the underlying molecular mechanisms remain unclear.

Network toxicology integrates protein-protein interaction networks, virtual docking, and topological analyses [9]. This approach enables the transformation of complex multi-target toxicity pathways into visually interpretable network models, which support systematic target identification and mechanistic interpretation of pathology induced by toxic substances. Network toxicology enables efficient, comprehensive, and systematically insightful analysis of toxic mechanisms, offering a global perspective that complements conventional toxicity assessment methods.

Our study aims to elucidate the toxicological characteristics of PSs (2,6-DTB, TBHQ, and UV-328) through multidisciplinary approaches, including network toxicology, machine learning, molecule docking, and MD simulations, and reveals that PSs may induce BC by regulating the MAPK14, PIM1, and TRDMT1 proteins. This study provides mechanistic insights into the safety evaluation of PSs through an interdisciplinary approach, thereby offering novel insights for the prevention and diagnosis of PS-induced breast cancer.

## 2. Methods

### 2.1. Study design and workflow

**Fig 1:**
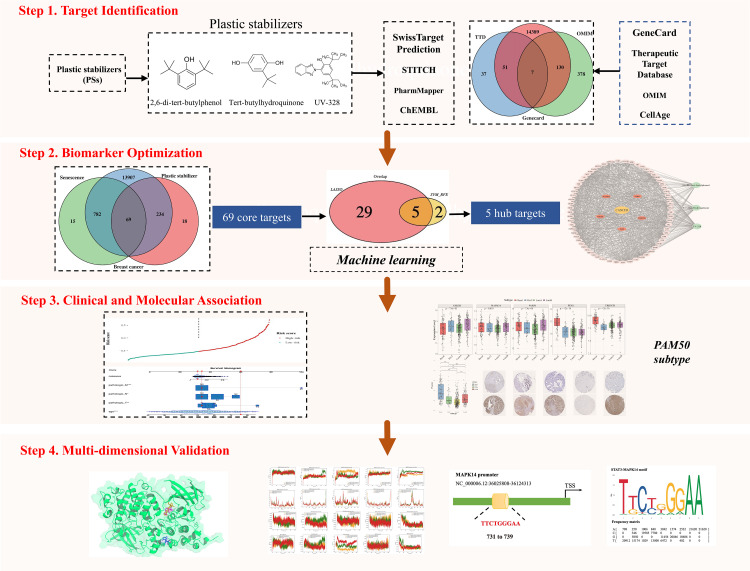
The schematic workflow of the study design. The study was conducted in four integrated phases: (1) **Target Identification**: Screening of potential targets for three representative plastic stabilizers (PSs: 2,6-DTB, TBHQ, UV-328) and their intersection with breast cancer- and senescence-related genes to identify disease-relevant targets; (2) **Biomarker Optimization**: Selection of robust diagnostic features using the intersection of LASSO and SVM-RFE machine learning algorithms; (3) **Clinical and Molecular Association**: Construction of a prognostic risk model, functional enrichment analysis (GO/KEGG/GSEA), and correlation analysis with PAM50 intrinsic subtypes; and (4) **Multi-dimensional Validation**: *In silico* verification of binding affinity via molecular docking and molecular dynamics (MD) simulations, followed by *in vitro* biological validation using breast cancer cell lines.

To systematically elucidate the potential carcinogenic mechanisms of PSs in breast cancer, we implemented a multi-stage integrative strategy (**Fig 1**). (1) Target identification: Initially, to define the biological scope of PS toxicity, we screened potential targets of three representative PSs (2,6-DTB, TBHQ, UV-328) and intersected them with breast cancer- and senescence-related genes. This filtration step was designed to exclude irrelevant targets and prioritize candidate genes specifically driving PS-induced pathogenesis. (2) Biomarker optimization: Recognizing the high dimensionality of genomic data, we applied two independent machine learning algorithms, LASSO and SVM-RFE, to mitigate overfitting and isolate the most robust prognostic features. The intersection of these approaches yielded a high-confidence consensus signature comprising 5 core genes. (3) Clinical and molecular association: A prognostic risk model was subsequently constructed and validated for predictive accuracy. Moreover, given the molecular heterogeneity of breast cancer, we analyzed the association between this signature and PAM50 intrinsic subtypes to pinpoint patient subgroups specifically susceptible to PS exposure. (4) Multi-dimensional validation: Finally, to bridge computational predictions with biological reality, the interactions between PSs and core targets were verified in silico via molecular docking and molecular dynamics (MD) simulations, and further confirmed in vitro through cell proliferation and gene expression assays.

### 2.2. Information of chemical components in common PSs

The information on commonly used PSs was obtained from PubMed, Google Scholar, and PubChem databases.

### 2.3. Targets collection of PSs

We extracted SMILES structures of 2,6-DTB, TBHQ, and UV-328 from PubChem (https://pubchem.ncbi.nlm.nih.gov), and their potential target genes were collected from SwissTargetPrediction (http://www.swisstargetprediction.ch) [10], STITCH (http://stitch.embl.de/), ChEMBL [11] and PharmMapper (https://lilab-ecust.cn/pharmmapper/index.html) [12]. Target genes were subsequently filtered for human (*Homo sapiens*) orthologues only, and followed by further name standardization through the UniProt database.

### 2.4. Screening of disease-related targets

We obtained breast cancer-associated targets from GeneCards [13], Online Mendelian Inheritance in Man (OMIM) [14] and the Therapeutic Target Database (TTD) [15]. Senescence-related genes were extracted from the CellAge database [16] (https://genomics.senescence.info/cells).

### 2.5. Construction of protein–protein interaction (PPI) networks

We obtained the intersectional targets between disease and PSs using the VennDiagram package (v1.7.3) in R software (v4.4.0; R Foundation for Statistical Computing, 2024). These intersecting targets were then used to construct a PPI network using the STRING database and were subsequently visualized using Cytoscape software (v3.9.1) [17,18]. To validate the protein expression of the core genes, immunohistochemistry (IHC) staining images of breast cancer and normal tissues were retrieved from the Human Protein Atlas (HPA) database (https://www.proteinatlas.org).

### 2.6. GO and KEGG enrichment analysis

We performed Kyoto Encyclopedia of Genes and Genomes (KEGG) and Gene Ontology (GO) enrichment analyses on intersectional targets between disease and PSs using the R packages “*clusterProfiler*” and “*ggplot2*” [19], and visualized the top 15 terms from the GO [20] and KEGG analyses [21].

### 2.7. Construction of a diagnostic model and predictive value assessment

To eliminate overfitting and identify the most significant prognostic features, the 69 overlapping genes were input into the Least Absolute Shrinkage and Selection Operator (LASSO) regression model using the “glmnet” R package [22]. The LASSO algorithm shrinks the coefficients of irrelevant genes to zero by constructing a penalty function. We employed 10-fold cross-validation to tune the optimal penalty parameter (λ). The optimal λ value was selected based on the minimum partial likelihood deviance (lambda.min), and genes with non-zero coefficients at this value were identified as candidate markers.

Support Vector Machine-Recursive Feature Elimination (SVM-RFE) was applied as a complementary method to screen for the most robust feature subset with the highest discriminatory power [23,24]. Using the “e1071” and “caret” R packages, we performed feature ranking based on the weight vector magnitude. A 5-fold cross-validation was implemented to evaluate the classification performance of different gene subsets. The accuracy (or minimal RMSE) was used as the performance metric to determine the optimal number of features. The recursive process continued until the subset providing the highest classification accuracy was identified. We selected the top-ranked genes that contributed to the minimal error rate in the SVM-RFE model.

Finally, to ensure the reliability of the diagnostic model, we selected the overlapping genes identified by both LASSO and SVM-RFE algorithms as the final core diagnostic signature. The prognostic Risk Score was constructed based on the linear combination of the expression levels of the five core genes weighted by their respective regression coefficients. These coefficients were derived from a multivariate Cox proportional hazards regression analysis where all five genes were included in the model. The formula is defined as follows: Risk Score = $\sum_{i=1}^{5}\left(\beta i\;\times \text{ Exp}i\right)$. n = 5: The number of prognostic signature genes (TRDMT1, GSK3B, MAPK14, PIM1, PARP1). *βi*: The multivariate Cox regression coefficient for gene *i*. Exp_*i*_: The expression level of gene *i*. Patients were stratified into high- and low-risk groups based on the median value of the calculated risk scores. Kaplan–Meier (KM) analysis was conducted to evaluate the survival differences between the two groups using the “*survival*” and “*survminer*” R packages. Then, receiver operating characteristic (ROC) curves were used for assessing the predictive value of the risk model using the “*survivalROC*” package. Furthermore, independent datasets from TCGA and GEO (accession: GSE20685) were analyzed for external validation. Additionally, we constructed nomogram based on the risk signature and clinicopathologic characteristics including age, TNM stage, and decision curve analysis evaluated the diagnostic performance using the “*stdca.R*” script.

### 2.8. PAM50 classification with gene expression

To compare the classifications produced by our machine learning models with the standard PAM50 subtyping based on mRNA expression, we utilized breast cancer patient expression profiles from the TCGA-BRCA dataset, which were obtained through the UCSC Xena database (https://xenabrowser.net/datapages/). PAM50 subtype assignments (Luminal A, Luminal B, HER2-enriched, and Basal-like) were performed using the molecular.subtyping function within the genefu R package [25], based on the standard 50-gene signature algorithm described by Parker et al [26]. Subsequently, we conducted an analysis of the distribution of machine learning-derived PSs risk categories (high versus low) within each PAM50 subtype to assess their correlation.

### 2.9. Molecular docking

We obtained five protein crystal structures from the Protein Data Bank (PDB) [27], specifically GSK3B (1H8F), MAPK14 (1A9U), PARP1 (1UK0), PIM1 (1XQZ), and TRDMT1 (1G55). PyMOL was utilized to eliminate water molecules and original ligands from the target proteins. Subsequently, these proteins were imported into AutoDockTools 1.5.6 for hydrogenation, charge calculation, and the merging of non-polar hydrogens. The ligand molecules analyzed included 2,6-DTB, TBHQ, and UV-328, with their three-dimensional structures sourced from the PubChem database. The structures of the ligand molecules were optimized using OpenBabel 3.1.1 software and converted into pdbqt format with AutoDockTools 1.5.6 [28]. Docking grid settings of protein and small molecule are shown in **S1 File** **and** **S2 File**. Molecular docking between the ligands and the target proteins was performed using AutoDock Vina 1.2.3 software [29,30]. Finally, Discovery Studio 2019 and PyMOL were employed to generate comprehensive visualizations and conduct analyses of the protein-ligand interactions.

### 2.10 Molecular dynamics simulation

MD simulations were conducted on complexes involving the proteins GSK3B, MAPK14, PARP1, PIM1, and TRDMT1 with the ligands 2,6-DTB, TBHQ, and UV-328. All simulations were conducted using GROMACS 2025.1. The Amber99SB-ILDN force field was applied to the proteins, while the GAFF force field was employed for the compounds. Solvation was performed with the TIP3P water model within a periodic boundary box, maintaining a 1.0 nm margin. The system was neutralized using sodium and chloride ions. Prior to commencing the production runs, the systems underwent energy minimization and equilibration processes under the canonical ensemble (NVT) and the isothermal-isobaric ensemble (NPT). Subsequently, the resulting trajectories were subjected to analysis for root mean square deviation (RMSD), root mean square fluctuation (RMSF), radius of gyration (Rg), and the count of hydrogen bonds between proteins and ligands.

### 2.11. Cell Lines and reagents

Human breast cancer cell lines MDA-MB-231 and MCF7 were obtained from Procell Life Science & Technology Co., Ltd. (Wuhan, China). All cells were cultured in DMEM (Cytiva, USA) supplemented with 10% fetal bovine serum (FBS) (Life-iLab, Shanghai, China) and 1% penicillin-streptomycin at 37°C in a humidified incubator with 5% CO₂. The antioxidant tert-butylhydroquinone (TBHQ) and UV-328 were purchased from MedChemExpress (Cat# HY-100489) and Sigma-Aldrich (Cat# 535753), respectively. Primary antibodies against MAPK14 (Cat# 14064–1-AP), PIM1 (Cat# 27196–1-AP), and TRDMT1 (Cat# 19221–1-AP) were purchased from Proteintech (USA). Antibodies targeting STAT3 (Cat# 9139) and Phospho-STAT3 (Tyr705) (Cat# 9145) were obtained from Cell Signaling Technology (USA). The anti-β-actin antibody (Cat# AC038) was acquired from ABclonal (Wuhan, China).

### 2.12. Cell proliferation assay

Cell viability was assessed using the Cell Counting Kit-8 (CCK-8) assay. Human breast cancer cell lines, MDA-MB-231 and MCF7, were seeded into 96-well plates at a density of 2,000–3,000 cells per well. After cell attachment, the cells were treated with DMSO (as a control), TBHQ, or UV-328 at indicated concentrations. At the specified time points, the original medium was replaced with a culture medium containing 10% CCK-8 reagent (Cat#C0005, TargetMol, USA). Following a 2-hour incubation period in a humidified incubator at 37°C, the absorbance at 450 nm was measured using a microplate reader to determine cell viability. All experiments were performed in at least three independent biological replicates.

### 2.13. RNA Extraction, Reverse Transcription, and Quantitative Real-Time PCR (RT-qPCR)

RNA was extracted from cultured cells using RNA-easy isolation reagent (#R701-01; Vazyme). Next, cDNA was prepared using HiScript II Q RT SuperMix (Vazyme; R223-01), which was assayed for qRT-PCR using ChamQ Universal SYBR qPCR Master Mix (#Q711-02; Vazyme). The used primers were as follows:

*GSK3B*-F: TTGGAGCCACTGATTACACG;

*GSK3B*-R: CCAACTGATCCACACCACTG;

*MAPK14*-F: GACCGTTTCAGTCCATCATTCA;

*MAPK14*-R: CTGGCACTTCACGATGTTGTTC;

*PARP1*-F: CTGGGGAGTCGGCGATCTT;

*PARP1*-R: GGTTACCCACTCCTTCCGGT;

*PIM1*-F: GCTCGGTCTACTCTGGCATC;

*PIM1*-R: CCGAGCTCACCTTCTTCAAC;

*TRDMT1*-F: TCTCCAACCTCTCTTGGCATTC;

*TRDMT1*-R: GGGAACTCCATCAGTACCTGACCA.

### 2.14. Wound healing assay

Cells were seeded in 6-well plates at 5–6 × 10⁵ cells per well, and were vertically scratched using a sterile 200 μl pipette tip when the cells reached 90–100% confluency. Then, the medium was replaced by low-serum DMEM (1% FBS) containing 5 μM TBHQ or 1μM UV-328 and DMSO after washing off the shed cells with phosphate-buffered saline (PBS). Finally, images at 0 h and 24 h were taken under a light microscope under × 10 lens in three random fields.

### 2.15. The Transcription Factor Target Finder (TFTF) analysis

ChIP-seq high-throughput data from *TFTF* package was employed for identifying transcription factors which was responsible for transcription of core target genes [31]. We intersected these transcription factors found for every hub gene across six databases (hTFtarget, KnockTF, CHEA, TRRUST, GTRD, and ChIP-Atlas) to determine the most potential transcription factor for each target gene. Then, we analyzed the potential TF-binding sites on hub gene promoter region (−2000 bp upstream) using the JASPAR database.

### 2.16. Statistical analysis

All quantitative data are presented as the mean ± SD. Statistical analyses were performed using R software (v4.4.0) and GraphPad Prism. The Shapiro-Wilk test was used to evaluate the normality of the data distribution. For normally distributed data, comparisons between two groups were performed using Student’s t-test, and multi-group comparisons were conducted using one-way ANOVA. For non-normally distributed data, the Wilcoxon rank-sum test or Kruskal-Wallis test was applied. Survival analysis was performed using the Kaplan-Meier method, and differences were determined by the log-rank test. Receiver operating characteristic (ROC) curves were generated to evaluate predictive accuracy. Statistical significance was set at *P* < 0.05, with non-significant results indicated as n.s. All in vitro experiments were conducted in three independent biological replicates.

## 3. Results

### 3.1. Chemical information of three PSs

**Fig 2:**
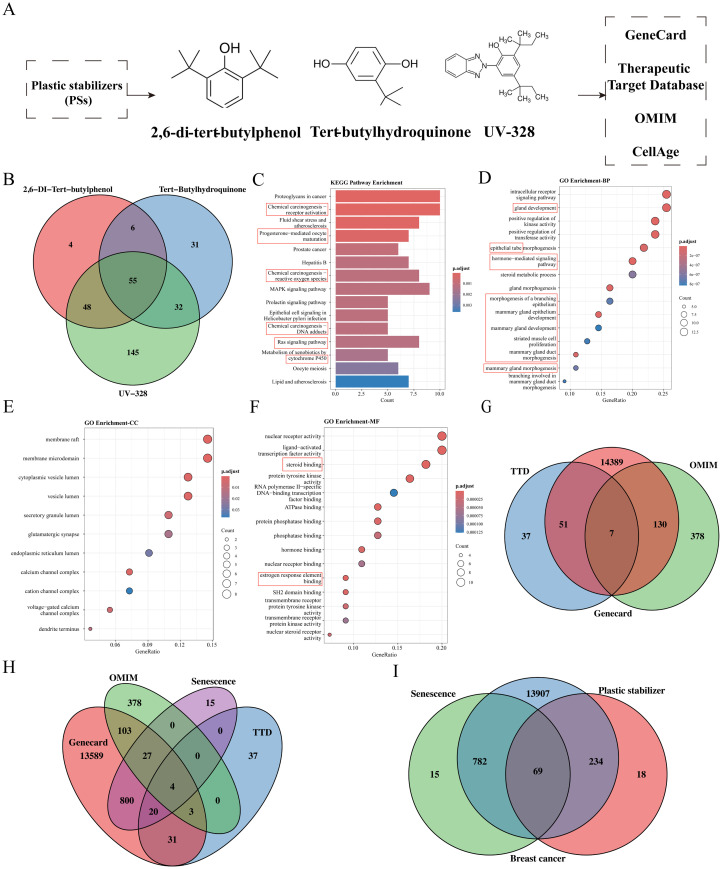
Identification of cancer targets associated with plastic stabilizers (PSs). **A:** Chemical structures of common PSs (2,6-DTB, TBHQ, UV-328) and the target screening strategy. **B:** Venn diagram of three PSs targets. **C-F:** KEGG and GO enrichment analyses of the 55 core genes associated with PS compounds. **G:** Venn diagram for breast cancer targets in three databases. **H:** Venn diagram of breast cancer and senescence targets. **I:** Venn diagram for breast cancer, senescence, and PS targets.

Considering the PS products that are presently predominant in China, the European Union, and the United States, we have identified three commonly utilized PSs: 2,6-DTB, TBHQ, and UV-328 (**Fig 2A**). The chemical formulas and SMILES structures of these three PSs are provided in **Table 1**.

**Table 1:** The active ingredients of plastic stabilizers and the molecular structure of target proteins.

PubChem CID	Compounds	Preferred IUPAC name	Chemical formula	SMILES structures
31405	2,6-DI-Tert-butylphenol	2,6-Di-tert-butylphenol	C_14_H_22_O	CC(C)(C)C1 = C(C(=CC = C1)C(C)(C)C)O
16043	Tert-Butylhydroquinone	2-tert-Butylbenzene-1,4-diol	C_10_H_14_O_2_	CC(C)(C)C1 = C(C = CC(=C1)O)O
33263	UV-328	2-(2H-1,2,3-Benzotriazol-2-yl)-4,6-bis(2-methylbutan-2-yl) phenol	C_22_H_29_N_3_O	CCC(C)(C)C1 = CC(=C(C(=C1)N2N=C3C = CC = CC3 = N2)O)C(C)(C)CC
**Targets**	**PDB ID**	**Method**	**Resolution**	**UniProt ID**
GSK3B	1H8F	X-RAY DIFFRACTION	2.80 Å	P49841
MAPK14	1A9U	X-RAY DIFFRACTION	2.50 Å	Q16539
PARP1	1UK0	X-RAY DIFFRACTION	3.00 Å	P09874
PIM1	1XQZ	X-RAY DIFFRACTION	2.10 Å	P11309
TRDMT1	1G55	X-RAY DIFFRACTION	1.80 Å	O14717

### 3.2. Target genes of the three PSs

A total of 3,121 protein targets were predicted for the three PSs. Specifically, 113, 124, and 280 unique targets were predicted for 2,6-DTB, TBHQ, and UV-328, respectively. The intersection of these three target sets resulted in 55 common targets (**Fig 2B**).

### 3.3. Core target gene functional enrichment analysis

The analysis identified cellular components (CC) and molecular functions (MF), with the top 15 most significantly enriched KEGG pathways highlighted for visualization. KEGG pathway analysis showed notable enrichment in cancer-related pathways, signal transduction, and metabolism, including chemical carcinogenesis, the MAPK and Ras signaling pathways, and the prolactin signaling pathway. The chemical carcinogenesis pathway was particularly prominent across multiple categories (**Fig 2C**). Bubble charts displayed the top 15 terms from each GO category (Fig 2D**–2F**). In BP, significant enrichment was identified in processes such as intracellular receptor signaling, hormone-mediated pathways, mammary epithelium development, and mammary gland development. MF analysis demonstrated enrichment in protein tyrosine kinase activity, hormone binding, and estrogen response element binding. In CC, critical components associated with cellular trafficking and signal transduction were recognized, including the cytoplasmic vesicle lumen and membrane rafts.

### 3.4. Identification of breast cancer-related target genes

The integration of target data from the GeneCard, OMIM, and TTD databases resulted in the identification of a total of 14,992 breast cancer-related target genes (**Fig 2G**). Given the significant enrichment of PSs in pathways related to senescence, as elucidated by KEGG and GO analyses, an additional retrieval of 866 senescence-associated target genes was conducted from the CellAge database. The findings demonstrated an overlap of 851 target proteins between breast cancer and senescence-related targets (**Fig 2H**). Moreover, the study identified 14,992 target genes linked to breast cancer, 866 genes associated with aging, and 321 genes related to PS, with 69 genes being common across these categories (**Fig 2I**). Consequently, these 69 overlapping genes may serve as potential targets through which PSs facilitate the progression of breast cancer.

### 3.5. Identification of diagnostic hub genes using machine learning

**Fig 3:**
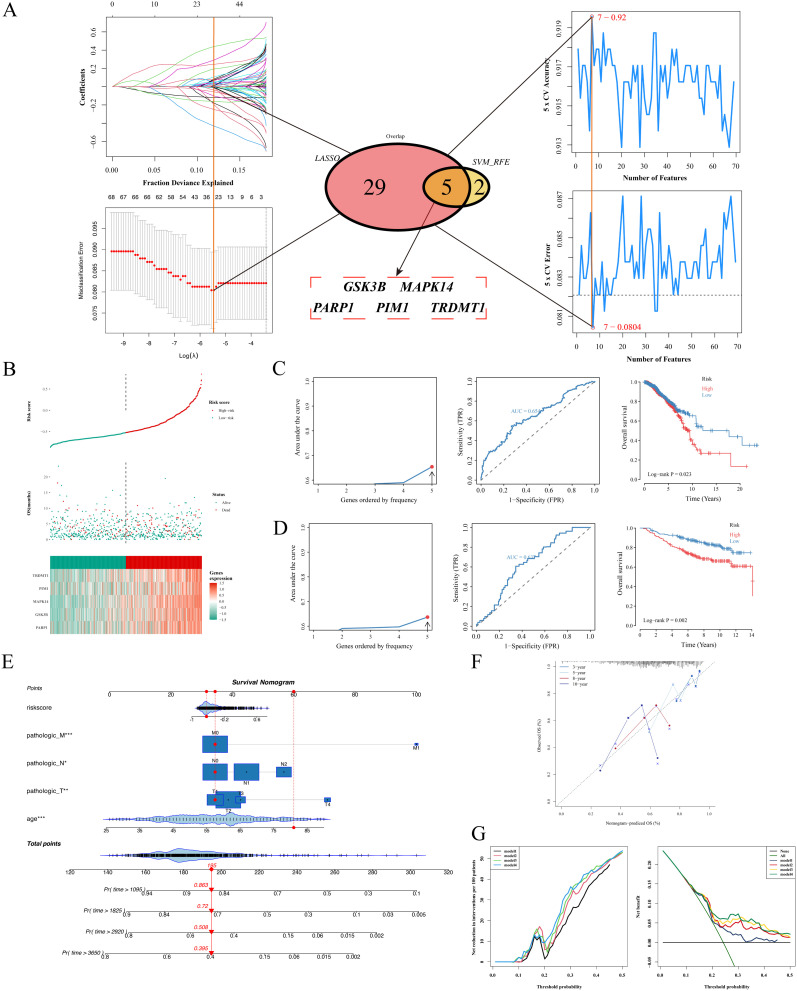
Construction and validation of the prognostic hub gene signature. **A:** Feature selection process using the Least Absolute Shrinkage and Selection Operator (LASSO) regression (left) and Support Vector Machine-Recursive Feature Elimination (SVM-RFE) algorithms (right) to screen for optimal biomarkers. **B:** Risk score distribution analysis in the training set. Upper panel: Distribution of risk scores (red: high risk; green: low risk). Middle panel: Survival status of patients (red: deceased; green: alive). Lower panel: Heatmap showing the expression profiles of the 5-gene signature. **C, D:** Assessment of the prognostic model in the training set (TCGA) and external validation set (GEO, GSE20685). Left: Risk score distribution and survival status. Middle: Receiver operating characteristic (ROC) curves with Area Under the Curve (AUC) values indicating predictive accuracy for 1-, 3-, and 5-year survival. Right: Kaplan-Meier survival analysis comparing Overall Survival (OS) between high- and low-risk groups. *P*-values were calculated using the log-rank test. **E:** Nomogram integrating the PS-related risk score with clinicopathological features (age, TNM stage) to predict 3-, 5-, 8-, and 10-year survival probabilities. **F:** Calibration curves showing the concordance between predicted survival probabilities (x-axis) and actual observed outcomes (y-axis). The diagonal line represents perfect prediction. **G:** Decision Curve Analysis (DCA) evaluating the net clinical benefit of different models. **Model 1:** risk score + age; **Model 2:** risk score + age + pathologic T stage; **Model 3:** risk score + age + pathologic T stage + pathologic N stage; **Model 4:** risk score + age + pathologic T stage + pathologic N stage + pathologic M stage.

*GSK3B*, *MAPK14*, *PARP1*, *PIM1*, and *TRDMT1* were consistently validated as an optimal diagnostic signature (**Fig 3A**). Based on risk scores, patients were classified into high- and low-risk groups. Survival analysis demonstrated significantly higher mortality in the high-risk group compared to the low-risk group (**Fig 3B**). The ROC curve analysis indicated that the gene signature exhibited moderate accuracy in both the training set (AUC: 0.654) (TCGA-BRCA; **Fig 3C**) and external validation sets (AUC: 0.637) (GSE20685) (**Fig 3D**).

We established a nomogram incorporating clinicopathological features, PSs risk score, and patient prognosis to facilitate quantitative assessment of breast cancer patient outcomes (**Fig 3E**). Using age, TNM stage, and risk scores, total scores were calculated to estimate 3-year, 5-year, 8-year, and 10-year survival probabilities. Calibration curves demonstrated a high correlation between the predicted survival rates derived from the nomogram and the observed survival rates (**Fig 3F**).

Model performance was evaluated through decision curve analysis (DCA), which revealed that Model 4 (risk score + age + pathologic T/N/M stages) offered optimal clinical utility, providing the highest net benefit across various threshold probabilities while notably reducing unnecessary interventions, particularly at higher risk thresholds. These results highlight that the incorporation of key clinical parameters, such as pathologic N/M stages, enhances decision-making accuracy and maximizes net benefit (**Fig 3G**). Collectively, these findings affirm the clinical utility of these models in guiding intervention strategies for high-risk patient populations.

### 3.6. PSs-driven breast carcinogenesis via five core gene modulations

**Fig 4:**
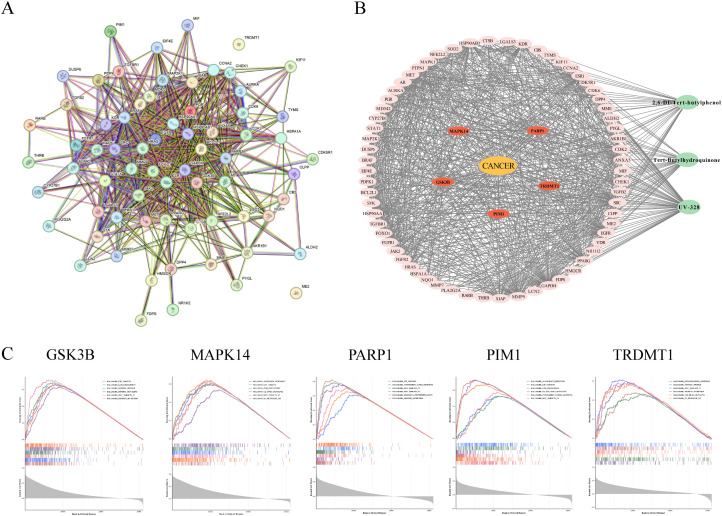
Protein-protein interaction (PPI) and functional enrichment networks. **A:** PPI network of the 69 overlapping genes constructed via the STRING database. Nodes represent proteins, and edges represent interactions. **B:** The PSs-Target-Cancer network. Green nodes: Plastic stabilizer compounds; Orange node: Breast cancer; Red nodes: The five core hub genes; Gray lines: Direct interactions between chemicals and gene targets. **C:** GSEA enrichment analysis map of 5 hub genes.

An intersection analysis of PS-associated genes and breast cancer senescence-related genes identified 69 overlapping genes, for which a protein-protein interaction (PPI) network was constructed (**Fig 4A**). A cancer-compound interaction network was also established for these 69 key genes (**Fig 4B**). Enrichment analysis of the five key genes revealed their predominant association with hallmark pathways, including HALLMARK MYC TARGETS, G2M CHECKPOINT, and E2F TARGETS. These pathways are functionally linked to tumor proliferation, metabolic reprogramming, and cell cycle regulation (**Fig 4C**). These findings show that PSs may help mammary cells evade apoptosis, bypass cell cycle checkpoints, promote uncontrolled growth, and drive breast cancer development.

### 3.7. Molecular docking of PSs to the core targets

**Fig 5:**
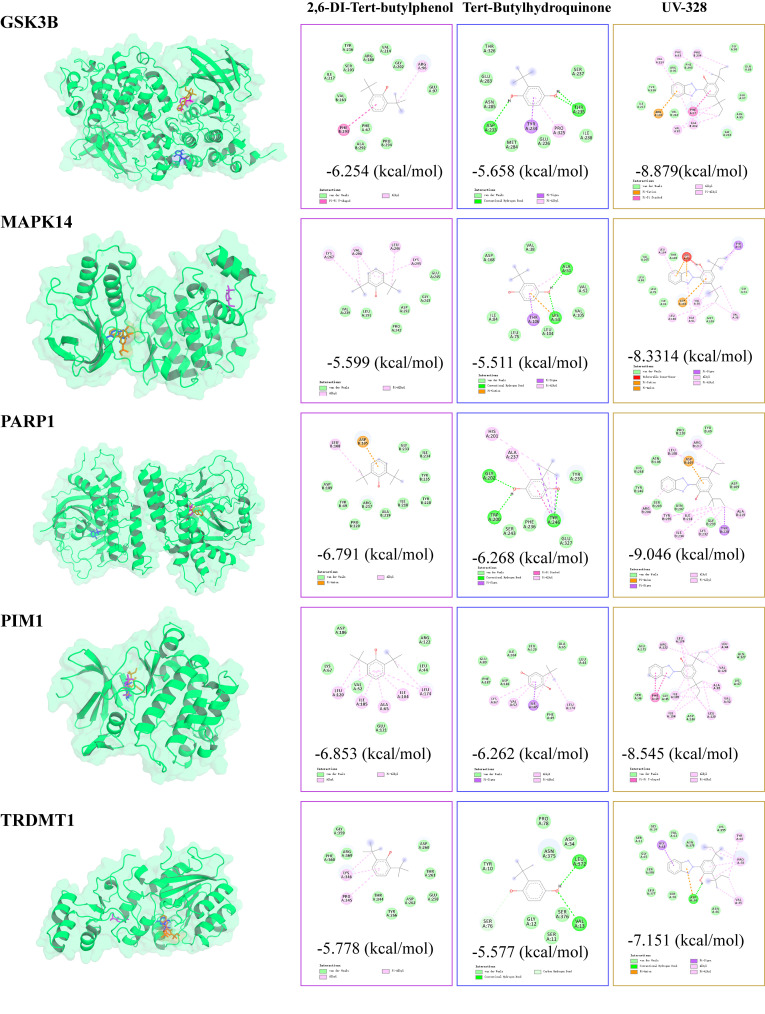
Molecular docking between plastic stabilizer (PSs) compounds and PSs-cancer hub targets. Visualization of the binding modes between three PSs (2,6-DTB, TBHQ, UV-328) and five core targets (GSK3B, MAPK14, PARP1, PIM1, TRDMT1). The 3D structures show the binding pockets, while 2D diagrams illustrate specific interaction types. The binding energies for the receptor-ligand complexes are provided in S2 File.

To assess the binding affinity of PSs for their respective targets, pairwise molecular docking analyses were conducted to evaluate the binding conformations and interactions between PSs and the five target proteins (**Fig 5** and **Table 1**). The findings indicated that all PS-protein complexes demonstrated binding energies below -5 kcal/mol, with TBHQ and UV-328 exhibiting a notably strong binding affinity to their targets.

### 3.8. Molecular dynamics (MD) simulation analysis

**Fig 6:**
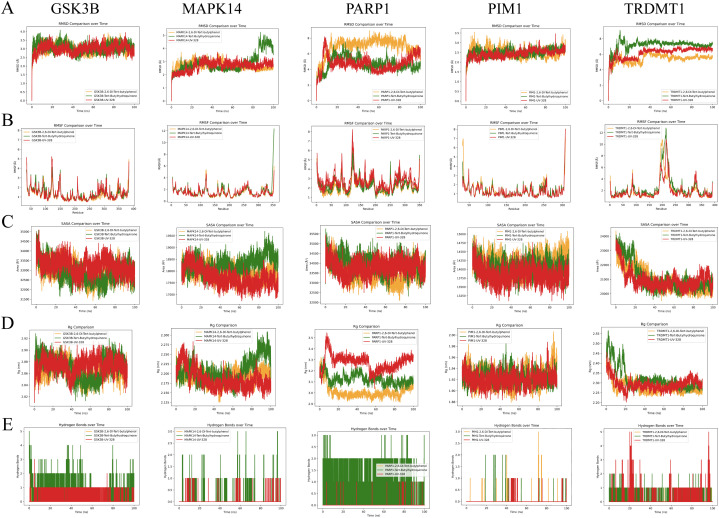
Molecular dynamics (MD) simulation analysis. **A:** Root Mean Square Deviation (RMSD): Trajectories showing the structural stability of the protein-ligand complexes over time. **B:** Root Mean Square Fluctuation (RMSF): Analysis of residue flexibility during the simulation, highlighting stable binding regions. **C:** Radius of Gyration (Rg): A measure of the compactness of the protein structure throughout the simulation. **D:** Hydrogen Bond Analysis: The number of hydrogen bonds formed between the ligands and proteins during the 100 ns simulation, indicating interaction stability.

We further validated the binding interactions and stability of the compounds with the respective proteins. MD simulations were conducted for 100 nanoseconds on complexes involving GSK3B, MAPK14, PARP1, PIM1, and TRDMT1 proteins with 2,6-DTB, TBHQ, and UV-328 (**Fig 6**). The RMSD trajectories of 2,6-DTB, TBHQ, and UV-328 complexes converged within 2 Å, indicating structural stability. (**Fig 6A**). This suggests strong binding stability across these systems. The MAPK14 complexes exhibited nearly overlapping RMSD curves (around 2 Å fluctuations) for 2,6-DTB and UV-328, indicating similar stabilization mechanisms. However, PARP1 complexes showed much more variation, with RMSD fluctuations exceeding 3 Å, which points to weaker binding stability and possible transient interactions (**Fig 6A**).

In order to further investigate the fluctuations observed in each amino acid residue within the protein during the simulation, we examined the Root Mean Square Fluctuation (RMSF) for all complexes. Elevated RMSF values denote increased flexibility in these regions. The PARP1 complexes demonstrated extensive flexibility across all residues, with a significant fluctuation peak exceeding 3.5 Å centered at residue 130. This indicates an inherent structural plasticity in PARP1, which may influence ligand binding. Conversely, the TRDMT1 complexes exhibited localized flexibility, particularly within residues 150–250, with consistent RMSF values around 2.8 Å. The limited mobility outside this domain suggests a domain-specific conformational adaptability (**Fig 6B**).

Analysis of the Solvent Accessible Surface Area (SASA) values revealed elevated exposure in GSK3B and PARP1 complexes, suggesting conformational changes. Such phenomena typically occur during protein unfolding and relaxation processes. Our findings indicated that the GSK3B and PARP1 proteins exhibited elevated SASA values, suggesting significant alterations attributable to surface exposure. These modifications are likely associated with conformational transitions, as well as folding or unfolding events in the proteins (**Fig 6C**).

PARP1 and TRDMT1 complexes exhibited larger gyration (Rg) values, indicating a more disordered or unfolded protein conformation, potentially indicative of partial unfolding or conformational relaxation. Furthermore, the variation in Rg values within the TRDMT1 group implies a conformational transition from a more relaxed to a more compact structure (**Fig 6D**). Finally, we quantified the number of hydrogen bonds formed within the protein and between the protein and ligands, as these interactions contribute to the stability and folded state of the protein-ligand complex. Notably, we found that TBHQ has the largest hydrogen bond counts over a 100-ns simulation period (**Fig 6E**).

### 3.9. PSs signature and PAM50 gene expression signatures subclassify

**Fig 7:**
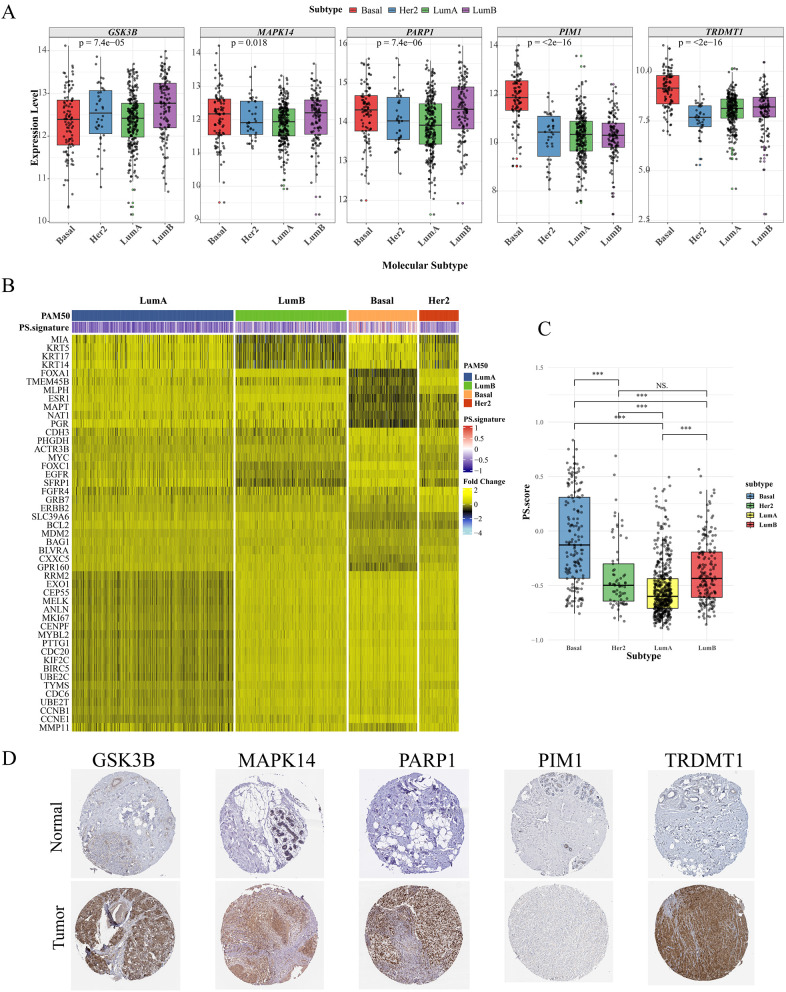
Association of the PSs signature with PAM50 intrinsic subtypes. **A:** Gene expression of five hub genes in different PAM50 subtypes (Luminal A, Luminal B, HER2, Basal-like). **B:** Differential distribution of risk scores across PAM50 subtypes. **C:** Boxplots illustrating the significant upregulation of core genes in specific subtypes. Statistical significance was determined using the Wilcoxon test (*P* < 0.05). **D:** Immunohistochemistry (IHC) validation of protein expression in breast cancer vs. normal tissues (Data from the Human Protein Atlas).

The classification of PAM50 is crucial in the subtyping of breast cancer, enabling more precise prognoses and customized treatment strategies. Initially, the expression patterns of five genes were analyzed across various molecular subtypes. Notably, *GSK3B* exhibited comparatively higher expression levels in the Lum B subtype, whereas *PIM1* and *TRDMT1* were upregulated in the basal subtype, suggesting that the PSs signature may primarily influence the progression of basal subtype breast cancer through *PIM1* and *TRDMT1* (**Fig 7A**). To clarify the relationship between PAM50 classification and the PSs signature, the expression levels of the gene set components associated with PAM50 were measured using RNA-seq data. The PAM50 algorithm was employed to stratify the TCGA-BRCA dataset into four subtypes: basal, Lum A, Lum B, and Her2 (**Fig 7B**).

Moreover, the basal subtype showed much higher PSs scores, whereas the Lum A subtype had the lowest levels of PSs (**Fig 7C**). Immunohistochemical analysis also indicated a significant increase in the proteins *GSK3B*, *MAPK14*, *PARP1*, *PIM1*, and *TRDMT1* in breast cancer tissues (**Fig 7D**).

### 3.10. PSs promote breast cancer cell proliferation by promoting MAPK14 through STAT3

**Fig 8:**
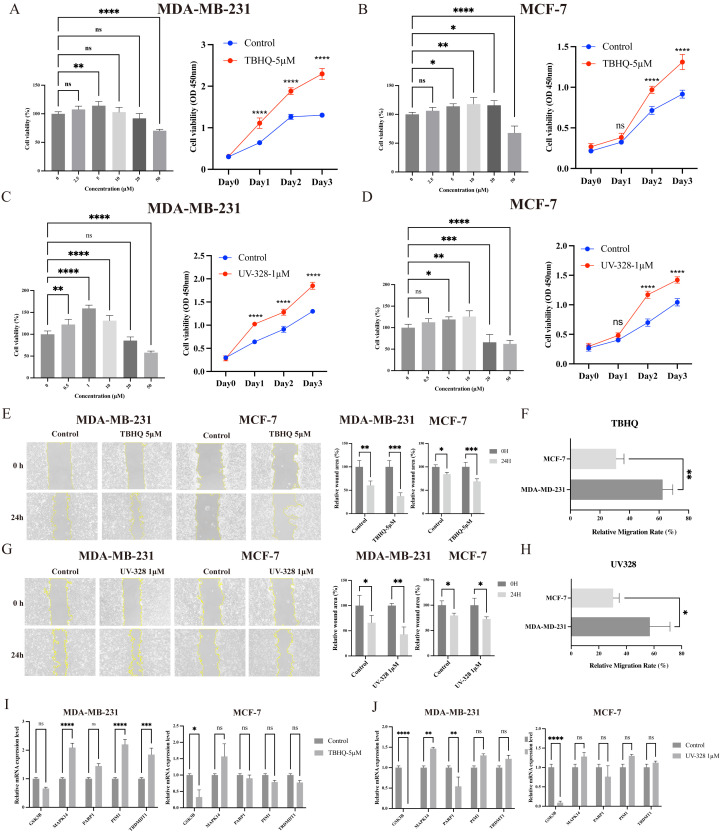
Plastic stabilizers (PSs) promote breast cancer progression. **A, B:** Effect of TBHQ on cell viability. Concentration- and time-dependent cell viability curves of two breast cancer cell lines treated with TBHQ (n = 3). **C, D:** Effect of UV-328 on cell viability. Concentration- and time-dependent cell viability curves of two breast cancer cell lines treated with another plastic stabilizer (PS) (n = 3). **E, G:** Wound healing assay. Representative images of wound healing assays in two breast cancer cell lines treated with TBHQ (E) or UV-328 **(G)** (n = 3). **F, H:** Quantification of cell migration. Quantitative analysis of the migration rates in two breast cancer cell lines following treatment with TBHQ (F) or UV-328 (H) compared to controls. **I, J:** RT-qPCR analysis. Relative mRNA expression levels of related markers in breast cancer cells (5 μM TBHQ or 1μM UV-328; 24h). Data are presented as mean ± SEM. Statistical significance versus controls is denoted as *p < 0.05, **p < 0.01, ***p < 0.001, ****p < 0.0001.

To investigate the impact of plastic stabilizers on cell growth, MDA-MB-231 and MCF-7 cell lines were treated with varying concentrations of TBHQ and UV328. Cell viability assays conducted 24 hours post-treatment revealed that low concentrations of both TBHQ (**Fig 8A and 8B**) and UV328 (**Fig 8C** **and 8D**) significantly facilitated breast cancer cell proliferation in a concentration- and time-dependent manner, whereas exposure to high doses exerted an inhibitory effect on cell viability. Consistent with this proliferative phenotype, wound healing assays demonstrated that cells treated with 5 μM TBHQ (**Fig 8E and 8F**) or 1 μM UV328 (**Fig 8G and 8H**) exhibited accelerated migration, resulting in faster wound closure rates compared to untreated controls. To further elucidate the molecular mechanisms underlying the pro-carcinogenic effects of PSs, we analyzed gene expression changes following treatment with TBHQ. A 24-hour exposure substantially elevated MAPK14 mRNA expression levels. Furthermore, upregulated transcripts of PIM1 and TRDMT1 were observed, particularly in MDA-MB-231 cells, reinforcing the evidence of the tumor-promoting potential of these stabilizers (**Fig 8I and 8J**).

**Fig 9:**
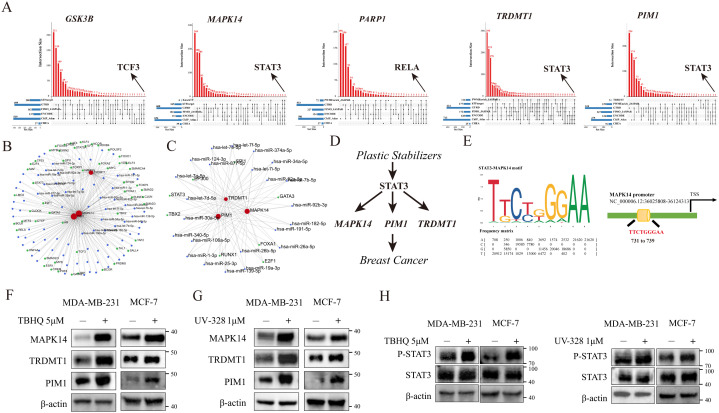
Exploration and experimental validation of the upstream regulatory mechanism. **A:** Venn diagram illustrating the intersection of potential upstream regulators (TFs) for the target genes identified by cross-referencing hTFtarget, GTRD, and ChIP-Atlas databases. **B:** Construction of the miRNA-TF-mRNA regulatory network comprising 144 TFs and 277 miRNAs. **C:** Identification of key transcription factors, highlighting STAT3, TBX2, EP300, RUNX1, E2F1, FOXA1, and GATA3 as central components. **D:** Schematic diagram illustrating the hypothesized signaling axis where PSs promote breast cancer progression via STAT3-mediated regulation. **E**: JASPAR motif analysis showing the high-affinity, conserved STAT3 binding site (TTCTGGGAA) within the MAPK14 promoter (relative score: 1.0). **F–H:** Western blot analysis validating the regulation of the identified targets by PSs.

Integrated analysis of ChIP-seq data from databases including hTFtarget, GTRD, and ChIP-Atlas identified upstream regulators of the target genes. Cross-referencing revealed that STAT3 consistently regulates MAPK14, PIM1, and TRDMT1, whereas GSK3B and PARP1 are controlled by TCF3 and RELA, respectively (**Fig 9A**). We subsequently constructed a miRNA-TF-mRNA network comprising 144 TFs and 277 miRNAs (**Fig 9B**), which highlighted STAT3, TBX2, EP300, RUNX1, E2F1, FOXA1, and GATA3 as key components of this regulatory axis (**Fig 9C**). These findings suggest that PSs promote breast cancer progression via STAT3-mediated regulation of MAPK14, PIM1, and TRDMT1 (**Fig 9D**). Furthermore, JASPAR motif analysis identified a high-affinity, conserved STAT3 binding site (TTCTGGGAA) within the MAPK14 promoter at positions 731–739 (relative score: 1.0), validating this direct regulatory interaction (**Fig 9E**). To further validate the regulatory role of PSs on these key targets at the protein level, Western blot analysis was performed (Fig 9F**-9H**). The results demonstrated that treatment with PSs significantly suppressed the protein expression levels of MAPK14, PIM1, and TRDMT1. Furthermore, activation of p-STAT3 was confirmed following PSs treatment. These experimental data robustly confirm STAT3 as a positive upstream regulator of this gene signature, thereby driving the oncogenic signaling axis.

## 4. Discussion

In this study, we integrated network toxicology, machine learning, and experimental validation to elucidate the molecular mechanisms linking plastic stabilizers (PSs) to breast cancer progression. We identified a core five-gene signature (*GSK3B*, *MAPK14*, *PARP1*, *PIM1*, and *TRDMT1*) associated with poor prognosis and validated that PSs promote tumorigenesis by upregulating *MAPK14*, *PIM1*, and *TRDMT1*, potentially via *STAT3*-mediated transcriptional regulation. Thus, our findings provide a new approach for evaluating the safety of plastic additives and offer novel toxicological insights into the role of PSs in breast cancer.

Plastic stabilizers are widely used in a range of products from personal care items to medical devices and food packaging to enhance the durability and extend the service life of plastic materials [32,1]. Owing to their extensive application and high lipophilicity, these compounds persistently accumulate in both humans and the environment, raising significant concerns about their potential health risks. Growing evidence have indicated that prolonged exposure to PSs may disrupt endocrine homeostasis, particularly in hormone-related function, potentially increasing disease risk [33,34]. However, no direct toxicological evidence is currently available due to the challenges in accurately quantifying exposure to PSs. We first provided important toxicological insights into the role of PSs in cancer risk. In this study, we found that PSs-associated genes are enriched in mammary epithelial development, gland morphogenesis, and estrogen signaling. Dysregulation of these processes may damage the integrity and function of the breast tissue and promote carcinogenesis. Additionally, these PSs-related genes are involved in the activation of the MYC, E2F, and G2/M signal pathways which regulate cell cycle and cellular senescence. These findings suggest that PSs may disrupt estrogen homeostasis and cell senescence, thereby increasing the risk of cancer development.

MAPK14 (p38α) is a key MAPK signaling component and regulates proliferation, migration, and apoptosis of cancer through the AKT/mTOR and PDK1 pathways [35]. PIM1 is a proto-oncogene that encodes a serine/threonine protein kinase, which potently promotes cell survival, proliferation, and metabolism by phosphorylating a series of downstream targets [36,37]. TRDMT1 primarily catalyzes the methylation of transfer RNA (tRNA) to stabilize RNA structure [38] [39]. Furthermore, it is highly expressed in various tumors and may promote tumor progression and therapy resistance by enhancing the stress adaptability and survival of cancer cells. These PS-associated hub genes in breast cancer were identified through machine learning optimization, and molecular docking and dynamics simulations suggested their strong interactions with PSs. STAT3 is a well-established proto-oncogene, and its constitutive activation is a hallmark of cancer, promoting multiple malignant behaviors in tumors including proliferation, angiogenesis, invasion, metastasis, and metabolic reprogramming [40,41]. Notably, TF regulatory analysis suggested that STAT3 is a key transcriptional regulator of MAPK14, PIM1, and TRDMT1. *In vitro,* PS upregulates these hub genes and the progression of breast cancer. Taken together, these data unveil that PSs potentially promote breast tumorigenesis via STAT3, which acts as a master upstream regulator to coordinately modulate the expression of MAPK14, PIM1, and TRDMT1. Crucially, the biological plausibility of this axis is supported by extensive literature confirming PIM1 as a direct target of STAT3 [42,43], which validates the accuracy of our predictive model. Building on this, our study proposes a novel mechanistic insight regarding the transcriptional control of MAPK14 and TRDMT1. Although direct chromatin binding assays were not performed in this study, the convergence of established signaling principles with our experimental observations strongly substantiates this coordinated regulatory network.

The PAM50 system facilitates precise molecular subtyping of breast cancer through gene expression profiling, classifying the disease into several distinct biological subtypes with varying therapeutic implications responses [44]. Specifically, the PAM50 classification includes basal-like, HER2-enriched, luminal A, and luminal B subtypes, among others. This precise stratification is essential not only for deepening our understanding of the complex heterogeneity of breast cancer but also plays an indispensable guiding role in clinical treatment decisions. Notably, the basal-like subtype has the worst prognosis of all types of breast cancer [45,46]. In this study, we uncovered for the first time a potential link between PSs and PAM50 subtyping. We found that five core genes were significantly upregulated in the basal-like subtype, and a PS-related risk score derived from these genes was highest in this subgroup. Therefore, the PS-associated gene signature may serve as a molecular biomarker, indicating that exposure to PSs may induce an elevated risk of developing the basal-like subtype. Given the distinct molecular features observed in basal-like tumors, we speculate that targeted reduction of PS exposure might confer clinical benefits to these patients. It is important to note that while our diagnostic model incorporates five core genes to maximize predictive accuracy, our investigation specifically focused on the MAPK14/PIM1/TRDMT1. This prioritization was driven by our multi-dimensional validation: molecular dynamics simulations indicated that these three targets formed more stable complexes with PSs compared to PARP1 and GSK3B, and they were identified to share a common transcriptional regulator, STAT3, suggesting a coordinated biological response to PS exposure. Together, inhibition of MAPK14, PIM1, and TRDMT1 may represent a promising subtype-specific therapeutic strategy, although this hypothesis warrants further experimental validation.

Crucially, our *in vitro* validation provided biological support for the subtype-specific risk predicted by our model. The inclusion of both MDA-MB-231 (Basal-like, high-risk) and MCF-7 (Luminal A, low-risk) cell lines allowed us to distinguish between generalized toxicity and subtype-specific molecular drivers. While plastic stabilizers promoted cell proliferation in both cell lines, illustrating a generalized toxicological risk, the underlying molecular responses exhibited distinct subtype specificity. Consistent with our PAM50 analysis, which showed intrinsic enrichment of MAPK14, PIM1 and TRDMT1 in Basal-like tumors, we observed a significantly more robust transcriptional upregulation of these two core targets in MDA-MB-231 cells following TBHQ exposure compared to MCF-7 cells. This dichotomy suggests a dual mechanism: while PS exposure is universally harmful, it may drive the poorer prognosis characteristic of Basal-like breast cancer specifically through the hyper-activation of the MAPK14/PIM1/TRDMT1 axis.

Our study had several limitations. First, the target analysis was primarily based on computational database predictions, which may not fully reflect biological contexts. Second, although we validated the findings through *in vitro* cellular experiments, the lack of *in vivo* animal models limits our ability to definitively establish a causal link regarding carcinogenesis. The simplified in vitro environment cannot fully replicate the complex systemic interactions and metabolic processes of the human body. Despite these limitations, our findings provide substantial evidence supporting the association between PSs and breast cancer pathogenesis. Future studies should incorporate multicenter clinical cohorts, epidemiological analyses, and single-cell spatial transcriptomic approaches to establish a comprehensive interdisciplinary validation framework.

## 5. Conclusion

This study identified five key molecular targets (GSK3B, MAPK14, PARP1, PIM1, and TRDMT1) that connect PSs to the progression of breast cancer, with their elevated expression associated with poorer prognostic outcomes. Moreover, PSs may facilitate breast cancer progression by activating the MYC, E2F, and G2/M pathways, disrupting estrogen homeostasis, and inducing breast cell senescence, thereby heightening the risk of cancer development. Mechanistically, PSs transcriptionally upregulate MAPK14, PIM1, and TRDMT1, with STAT3 mediating their transcriptional regulation. PSs also promote the proliferation and migration of breast cancer cells. The Basal-like subtype exhibited the highest risk score, suggesting a greater susceptibility to PSs. These results underscore the necessity of reassessing the safety of plastic additives and advocate for the adoption of more rigorous regulatory policies. Future investigations should concentrate on *in vivo* validation and epidemiological research to develop effective preventive strategies.

## Supporting information

S1 File Docking grid settings. (DOCX)

S2 File Docking affinity score. (DOCX)

## References

[1] Zhang S, Chen Y, Liu S, Li Y, Zhao H, Chen Q, et al. Dissolution-precipitation method concatenated sodium alginate/MOF-derived magnetic multistage pore carbon magnetic solid phase extraction for determination of antioxidants and ultraviolet stabilizers in polylactic acid food contact plastics. Talanta. 2024;270:125487. doi: 10.1016/j.talanta.2023.125487 38101034

[2] Wang X, Zhu L, Zhang J, Li D. Solvent extraction of UV stabilizers in plastics: A step towards methodology harmonization. Chemosphere. 2024;367:143594. doi: 10.1016/j.chemosphere.2024.143594 39442579

[3] Rioux B, Mouterde LMM, Alarcan J, Abiola TT, Vink MJA, Woolley JM, et al. An expeditive and green chemo-enzymatic route to diester sinapoyl-l-malate analogues: sustainable bioinspired and biosourced UV filters and molecular heaters. Chem Sci. 2023;14(47):13962–78. doi: 10.1039/d3sc04836e 38075651 PMC10699562

[4] Amadou A, Giampiccolo C, Bibi Ngaleu F, Praud D, Coudon T, Grassot L, et al. Multiple xenoestrogen air pollutants and breast cancer risk: Statistical approaches to investigate combined exposures effect. Environ Pollut. 2024;351:124043. doi: 10.1016/j.envpol.2024.124043 38679129

[5] White AJ, O’Brien KM, Niehoff NM, Carroll R, Sandler DP. Metallic Air Pollutants and Breast Cancer Risk in a Nationwide Cohort Study. Epidemiology. 2019;30(1):20–8. doi: 10.1097/EDE.0000000000000917 30198937 PMC6269205

[6] Wu AH, Wu J, Tseng C, Stram DO, Shariff-Marco S, Larson T, et al. Air Pollution and Breast Cancer Incidence in the Multiethnic Cohort Study. J Clin Oncol. 2025;43(3):273–84. doi: 10.1200/JCO.24.00418 39378392 PMC11735325

[7] Lee DG, Burstyn I, Lai AS, Grundy A, Friesen MC, Aronson KJ, et al. Women’s occupational exposure to polycyclic aromatic hydrocarbons and risk of breast cancer. Occup Environ Med. 2019;76(1):22–9. doi: 10.1136/oemed-2018-105261 30541747 PMC9366896

[8] Leng L, Li J, Luo X-M, Kim J-Y, Li Y-M, Guo X-M, et al. Polychlorinated biphenyls and breast cancer: A congener-specific meta-analysis. Environ Int. 2016;88:133–41. doi: 10.1016/j.envint.2015.12.022 26735351

[9] Yuan B, Li Y, Chang J, Guo C, Huang W, Wang Y. Molecular mechanism of bisphenols induction of breast cancer through PGR revealed by network toxicology and transcriptomics integration analysis. Ecotoxicol Environ Saf. 2025;300:118480. doi: 10.1016/j.ecoenv.2025.118480 40482448

[10] Daina A, Michielin O, Zoete V. SwissTargetPrediction: updated data and new features for efficient prediction of protein targets of small molecules. Nucleic Acids Res. 2019;47(W1):W357–64. doi: 10.1093/nar/gkz382 31106366 PMC6602486

[11] Zdrazil B, Felix E, Hunter F, Manners EJ, Blackshaw J, Corbett S, et al. The ChEMBL Database in 2023: a drug discovery platform spanning multiple bioactivity data types and time periods. Nucleic Acids Research. 2023;52(D1):D1180–92. doi: 10.1093/nar/gkad1004 PMC10767899 37933841

[12] Wang X, Shen Y, Wang S, Li S, Zhang W, Liu X, et al. PharmMapper 2017 update: a web server for potential drug target identification with a comprehensive target pharmacophore database. Nucleic Acids Res. 2017;45(W1):W356–60. doi: 10.1093/nar/gkx374 28472422 PMC5793840

[13] Stelzer G, Rosen N, Plaschkes I, Zimmerman S, Twik M, Fishilevich S, et al. The GeneCards Suite: From Gene Data Mining to Disease Genome Sequence Analyses. Curr Protoc Bioinformatics. 2016;54:1.30.1-1.30.33. doi: 10.1002/cpbi.5 27322403

[14] Hamosh A, Scott AF, Amberger JS, Bocchini CA, McKusick VA. Online Mendelian Inheritance in Man (OMIM), a knowledgebase of human genes and genetic disorders. Nucleic Acids Res. 2005;33(Database issue):D514-7. doi: 10.1093/nar/gki033 15608251 PMC539987

[15] Zhou Y, Zhang Y, Lian X, Li F, Wang C, Zhu F, et al. Therapeutic target database update 2022: facilitating drug discovery with enriched comparative data of targeted agents. Nucleic Acids Res. 2022;50(D1):D1398–407. doi: 10.1093/nar/gkab953 34718717 PMC8728281

[16] de Magalhães JP, Abidi Z, Dos Santos GA, Avelar RA, Barardo D, Chatsirisupachai K, et al. Human Ageing Genomic Resources: updates on key databases in ageing research. Nucleic Acids Res. 2024;52(D1):D900–8. doi: 10.1093/nar/gkad927 37933854 PMC10767973

[17] Shannon P, Markiel A, Ozier O, Baliga NS, Wang JT, Ramage D, et al. Cytoscape: a software environment for integrated models of biomolecular interaction networks. Genome Res. 2003;13(11):2498–504. doi: 10.1101/gr.1239303 14597658 PMC403769

[18] Szklarczyk D, Kirsch R, Koutrouli M, Nastou K, Mehryary F, Hachilif R, et al. The STRING database in 2023: protein-protein association networks and functional enrichment analyses for any sequenced genome of interest. Nucleic Acids Res. 2023;51(D1):D638–46. doi: 10.1093/nar/gkac1000 36370105 PMC9825434

[19] Wu T, Hu E, Xu S, Chen M, Guo P, Dai Z, et al. clusterProfiler 4.0: A universal enrichment tool for interpreting omics data. Innovation (Camb). 2021;2(3):100141. doi: 10.1016/j.xinn.2021.100141 34557778 PMC8454663

[20] Gene Ontology Consortium, Aleksander SA, Balhoff J, Carbon S, Cherry JM, Drabkin HJ, et al. The Gene Ontology knowledgebase in 2023. Genetics. 2023;224(1):iyad031. doi: 10.1093/genetics/iyad031 36866529 PMC10158837

[21] Kanehisa M, Furumichi M, Sato Y, Matsuura Y, Ishiguro-Watanabe M. KEGG: biological systems database as a model of the real world. Nucleic Acids Res. 2025;53(D1):D672–7. doi: 10.1093/nar/gkae909 39417505 PMC11701520

[22] Tibshirani R. Regression Shrinkage and Selection Via the Lasso. Journal of the Royal Statistical Society Series B: Statistical Methodology. 1996;58(1):267–88. doi: 10.1111/j.2517-6161.1996.tb02080.x

[23] Huang M-L, Hung Y-H, Lee WM, Li RK, Jiang B-R. SVM-RFE based feature selection and Taguchi parameters optimization for multiclass SVM classifier. ScientificWorldJournal. 2014;2014:795624. doi: 10.1155/2014/795624 25295306 PMC4175386

[24] Guo P, Luo Y, Mai G, Zhang M, Wang G, Zhao M, et al. Gene expression profile based classification models of psoriasis. Genomics. 2014;103(1):48–55. doi: 10.1016/j.ygeno.2013.11.001 24239985

[25] Gendoo DMA, Ratanasirigulchai N, Schröder MS, Paré L, Parker JS, Prat A, et al. Genefu: an R/Bioconductor package for computation of gene expression-based signatures in breast cancer. Bioinformatics. 2016;32(7):1097–9. doi: 10.1093/bioinformatics/btv693 26607490 PMC6410906

[26] Parker JS, Mullins M, Cheang MCU, Leung S, Voduc D, Vickery T, et al. Supervised risk predictor of breast cancer based on intrinsic subtypes. J Clin Oncol. 2009;27(8):1160–7. doi: 10.1200/JCO.2008.18.1370 19204204 PMC2667820

[27] Berman HM, Westbrook J, Feng Z, Gilliland G, Bhat TN, Weissig H, et al. The Protein Data Bank. Nucleic Acids Res. 2000;28(1):235–42. doi: 10.1093/nar/28.1.235 10592235 PMC102472

[28] Morris GM, Huey R, Lindstrom W, Sanner MF, Belew RK, Goodsell DS, et al. AutoDock4 and AutoDockTools4: Automated docking with selective receptor flexibility. J Comput Chem. 2009;30(16):2785–91. doi: 10.1002/jcc.21256 19399780 PMC2760638

[29] Trott O, Olson AJ. AutoDock Vina: improving the speed and accuracy of docking with a new scoring function, efficient optimization, and multithreading. J Comput Chem. 2010;31(2):455–61. doi: 10.1002/jcc.21334 19499576 PMC3041641

[30] Eberhardt J, Santos-Martins D, Tillack AF, Forli S. AutoDock Vina 1.2.0: New Docking Methods, Expanded Force Field, and Python Bindings. J Chem Inf Model. 2021;61(8):3891–8. doi: 10.1021/acs.jcim.1c00203 34278794 PMC10683950

[31] Wang J. TFTF: An R-Based Integrative Tool for Decoding Human Transcription Factor-Target Interactions. Biomolecules. 2024;14(7):749. doi: 10.3390/biom14070749 39062464 PMC11274450

[32] Sendra M, Pereiro P, Figueras A, Novoa B. An integrative toxicogenomic analysis of plastic additives. J Hazard Mater. 2021;409:124975. doi: 10.1016/j.jhazmat.2020.124975 33388451

[33] Hu Y, Jiang S, Zhang Q, Zhou W, Liang J, Xu Y, et al. Protective effect of Cordycepin on blood-testis barrier against pre-puberty polystyrene nanoplastics exposure in male rats. Part Fibre Toxicol. 2024;21(1):30. doi: 10.1186/s12989-024-00590-w 39118174 PMC11312894

[34] Jin H, Ding J, Liu H, Yang L, Li D, Han X. Chronic exposure to polystyrene microplastics induced LHR reduction and decreased testosterone levels through NF-κB pathway. Environ Pollut. 2024;358:124543. doi: 10.1016/j.envpol.2024.124543 39004204

[35] Zhao M, Zhou L, Zhang Q, Wang M, Dong Y, Wang Y, et al. Targeting MAPK14 by Lobeline Upregulates Slurp1-Mediated Inhibition of Alternative Activation of TAM and Retards Colorectal Cancer Growth. Adv Sci (Weinh). 2025;12(10):e2407900. doi: 10.1002/advs.202407900 39840525 PMC11904982

[36] Choudhury R, Bahadi CK, Ray IP, Dash P, Pattanaik I, Mishra S, et al. PIM1 kinase and its diverse substrate in solid tumors. Cell Commun Signal. 2024;22(1):529. doi: 10.1186/s12964-024-01898-y 39487435 PMC11531143

[37] Zhao G, Ren Y, Yan J, Zhang T, Lu P, Lei J, et al. Neoprzewaquinone A Inhibits Breast Cancer Cell Migration and Promotes Smooth Muscle Relaxation by Targeting PIM1 to Block ROCK2/STAT3 Pathway. Int J Mol Sci. 2023;24(6):5464. doi: 10.3390/ijms24065464 36982538 PMC10051292

[38] Chen H, Yang H, Zhu X, Yadav T, Ouyang J, Truesdell SS, et al. m5C modification of mRNA serves a DNA damage code to promote homologous recombination. Nat Commun. 2020;11(1):2834. doi: 10.1038/s41467-020-16722-7 32503981 PMC7275041

[39] Lewinska A, Adamczyk-Grochala J, Wnuk M. TRDMT1-mediated RNA C-5 methylation as a novel target in anticancer therapy. Biochim Biophys Acta Rev Cancer. 2023;1878(6):188964. doi: 10.1016/j.bbcan.2023.188964 37625528

[40] Jiang R-Y, Zhu J-Y, Zhang H-P, Yu Y, Dong Z-X, Zhou H-H, et al. STAT3: Key targets of growth-promoting receptor positive breast cancer. Cancer Cell Int. 2024;24(1):356. doi: 10.1186/s12935-024-03541-9 39468521 PMC11520424

[41] Hu Y, Dong Z, Liu K. Unraveling the complexity of STAT3 in cancer: molecular understanding and drug discovery. J Exp Clin Cancer Res. 2024;43(1):23. doi: 10.1186/s13046-024-02949-5 38245798 PMC10799433

[42] Gao X, Liu X, Lu Y, Wang Y, Cao W, Liu X, et al. PIM1 is responsible for IL-6-induced breast cancer cell EMT and stemness via c-myc activation. Breast Cancer. 2019;26(5):663–71. doi: 10.1007/s12282-019-00966-3 30989585 PMC6694096

[43] Li Q, Chen L, Luo C, ChenYan GJ, Zhu Z, et al. TAB3 upregulates PIM1 expression by directly activating the TAK1-STAT3 complex to promote colorectal cancer growth. Exp Cell Res. 2020;391(1):111975. doi: 10.1016/j.yexcr.2020.111975 32229191

[44] Suurmeijer JA, Soer EC, Dings MPG, Kim Y, Strijker M, Bonsing BA, et al. Impact of classical and basal-like molecular subtypes on overall survival in resected pancreatic cancer in the SPACIOUS-2 multicentre study. Br J Surg. 2022;109(11):1150–5. doi: 10.1093/bjs/znac272 35979597 PMC10364758

[45] Foulkes WD, Smith IE, Reis-Filho JS. Triple-negative breast cancer. N Engl J Med. 2010;363(20):1938–48. doi: 10.1056/NEJMra1001389 21067385

[46] Kennecke H, Yerushalmi R, Woods R, Cheang MCU, Voduc D, Speers CH, et al. Metastatic behavior of breast cancer subtypes. J Clin Oncol. 2010;28(20):3271–7. doi: 10.1200/JCO.2009.25.9820 20498394

