# Supplementary material for: An integrative analysis reveals the mechanism of plastic stabilizers inducing breast cancer
Source: PLoS Comput Biol. 2026 Mar 6;22(3):e1014025. doi: 10.1371/journal.pcbi.1014025 (PMC12965615; doi:10.1371/journal.pcbi.1014025)
Supplement: S1 File — (DOCX) [file pcbi.1014025.s001.docx]

**S1 material. Docking grid settings**

**GSK3B:**

center_x = 28.1395

center_y = -2.59

center_z = 23.803

size_x = 80.165

size_y = 89.218

size_z = 111.264

**MAPK14:**

center_x = 1.521

center_y = 20.841

center_z = 35.906

size_x = 67.119

size_y = 68.704

size_z = 69.282

**PARP1:**

center_x = 16.055

center_y = 15.804

center_z = 16.553

size_x = 80.672

size_y = 99.471

size_z = 95.014

**PIM1:**

center_x = 67.8295

center_y = 27.2045

center_z = -1.4215

size_x = 65.507

size_y = 62.5810

size_z = 56.3490

**TRDMT1:**

center_x = 66.4055

center_y = 41.4435

center_z = 41.5095

size_x = 58.467

size_y = 52.715

size_z = 85.201
