# Supplementary material for: An integrative analysis reveals the mechanism of plastic stabilizers inducing breast cancer
Source: PLoS Comput Biol. 2026 Mar 6;22(3):e1014025. doi: 10.1371/journal.pcbi.1014025 (PMC12965615; doi:10.1371/journal.pcbi.1014025)
Supplement: S2 File — (DOCX) [file pcbi.1014025.s002.docx]

**S2 material. Docking affinity score.**

**GSK3B:**

***2,6-DI-Tert-butylphenol***

mode | affinity | dist from best mode

| (kcal/mol) | rmsd l.b.| rmsd u.b.

-----+------------+----------+----------

1 -6.254 0 0

2 -6.24 33.66 36.63

3 -6.136 4.454 6.731

4 -5.794 36.93 38.94

5 -5.784 15.74 17.1

6 -5.778 35.64 38.76

7 -5.777 36.82 38.9

8 -5.761 35.64 38.67

9 -5.755 15.78 17.39

***Tert-Butylhydroquinone***

mode | affinity | dist from best mode

| (kcal/mol) | rmsd l.b.| rmsd u.b.

-----+------------+----------+----------

1 -5.658 0 0

2 -5.561 25.27 27.05

3 -5.477 26.72 28.42

4 -5.472 25.43 27.29

5 -5.445 24.59 26.06

6 -5.435 26.26 28.08

7 -5.427 25.6 27.25

8 -5.405 56.11 57.96

9 -5.297 25.77 27.23

***UV-328***

mode | affinity | dist from best mode

| (kcal/mol) | rmsd l.b.| rmsd u.b.

-----+------------+----------+----------

1 -8.879 0 0

2 -8.823 30.98 36.47

3 -8.565 36.77 39.16

4 -8.54 3.404 6.548

5 -8.514 1.992 6.12

6 -8.487 1.891 5.779

7 -8.017 2.079 6.258

8 -7.812 2.619 5.786

9 -7.58 4.321 8.338

**MAPK14:**

***2,6-DI-Tert-butylphenol***

mode | affinity | dist from best mode

| (kcal/mol) | rmsd l.b.| rmsd u.b.

-----+------------+----------+----------

1 -5.599 0 0

2 -5.581 0.08255 4.581

3 -5.421 1.986 3.795

4 -5.42 1.98 5.294

5 -5.396 35.27 37.15

6 -5.366 35.25 36.91

7 -5.275 35.85 37.58

8 -5.274 35.79 37.74

9 -5.2 34.26 36.33

***Tert-Butylhydroquinone***

mode | affinity | dist from best mode

| (kcal/mol) | rmsd l.b.| rmsd u.b.

-----+------------+----------+----------

1 -5.511 0 0

2 -5.307 6.466 7.928

3 -5.209 6.249 7.633

4 -5.209 1.291 3.263

5 -5.201 13.99 15.41

6 -5.134 36.01 37.08

7 -5.104 12.22 13.17

8 -5.091 36.57 38.4

9 -5.017 1.836 2.731

***UV-328***

mode | affinity | dist from best mode

| (kcal/mol) | rmsd l.b.| rmsd u.b.

-----+------------+----------+----------

1 -8.331 0 0

2 -7.931 2.331 6.047

3 -7.666 2.142 5.858

4 -7.627 1.132 4.431

5 -7.544 3.943 6.587

6 -7.495 29.58 32.22

7 -7.446 5.684 8.932

8 -7.22 3.446 7.023

9 -7.209 29.3 31.88

**PARP1:**

***2,6-DI-Tert-butylphenol***

mode | affinity | dist from best mode

| (kcal/mol) | rmsd l.b.| rmsd u.b.

-----+------------+----------+----------

1 -6.791 0 0

2 -6.762 0.1122 4.635

3 -6.618 41.14 43.27

4 -6.603 40.12 42.35

5 -6.586 40.02 42.5

6 -6.565 1.823 3.621

7 -6.561 1.793 5.451

8 -6.542 35.67 38.18

9 -6.514 35.69 37.93

***Tert-Butylhydroquinone***

mode | affinity | dist from best mode

| (kcal/mol) | rmsd l.b.| rmsd u.b.

-----+------------+----------+----------

1 -6.268 0 0

2 -6.13 8.194 10.38

3 -6.123 8.17 10.35

4 -6.055 49.92 52.3

5 -5.977 7.825 10.19

6 -5.869 7.201 9.579

7 -5.864 41.82 44.1

8 -5.821 52.17 53.82

9 -5.816 43.08 45.31

***UV-328***

mode | affinity | dist from best mode

| (kcal/mol) | rmsd l.b.| rmsd u.b.

-----+------------+----------+----------

1 -9.046 0 0

2 -8.857 3.426 6.213

3 -8.733 40.56 44.23

4 -8.197 38.69 42.34

5 -7.988 18.4 20.46

6 -7.905 19.42 22.51

7 -7.727 3.659 6.349

8 -7.606 19.04 20.68

9 -7.559 18.48 20.76

**PIM1:**

***2,6-DI-Tert-butylphenol***

mode | affinity | dist from best mode

| (kcal/mol) | rmsd l.b.| rmsd u.b.

-----+------------+----------+----------

1 -6.853 0 0

2 -6.848 0.07112 4.636

3 -6.699 2.719 4.541

4 -6.693 2.721 4.627

5 -6.544 1.408 5.004

6 -6.529 2.26 4.432

7 -6.5 2.249 3.503

8 -6.409 1.891 5.287

9 -6.346 1.643 5.383

***Tert-Butylhydroquinone***

mode | affinity | dist from best mode

| (kcal/mol) | rmsd l.b.| rmsd u.b.

-----+------------+----------+----------

1 -6.262 0 0

2 -6.144 1.727 2.506

3 -6.089 1.721 2.499

4 -5.891 11.68 13.12

5 -5.844 12.25 13.6

6 -5.776 2.119 3.281

7 -5.731 16.83 18.35

8 -5.673 1.545 3.367

9 -5.444 2.456 5.128

***UV-328***

mode | affinity | dist from best mode

| (kcal/mol) | rmsd l.b.| rmsd u.b.

-----+------------+----------+----------

1 -8.545 0 0

2 -8.455 1.048 4.644

3 -8.101 2.321 6.64

4 -7.866 2.082 6.007

5 -7.808 1.407 4.695

6 -7.749 1.449 4.67

7 -7.553 1.29 2.311

8 -7.33 1.962 6.241

9 -7.153 3.308 7.231

**TRDMT1:**

***2,6-DI-Tert-butylphenol***

mode | affinity | dist from best mode

| (kcal/mol) | rmsd l.b.| rmsd u.b.

-----+------------+----------+----------

1 -5.778 0 0

2 -5.763 0.04239 4.636

3 -5.544 1.457 2.843

4 -5.537 1.475 4.95

5 -5.401 10.45 12.53

6 -5.348 10.45 12.01

7 -5.342 1.335 4.768

8 -5.308 1.32 2.207

9 -5.188 20.65 22.56

***Tert-Butylhydroquinone***

mode | affinity | dist from best mode

| (kcal/mol) | rmsd l.b.| rmsd u.b.

-----+------------+----------+----------

1 -5.577 0 0

2 -5.507 6.771 8.682

3 -5.39 6.116 8.854

4 -5.327 18.42 20.44

5 -5.313 1.74 2.778

6 -5.167 18.88 20.59

7 -5.158 19.1 20.46

8 -5.135 16.1 18.78

9 -5.125 7.148 8.932

***UV-328***

mode | affinity | dist from best mode

| (kcal/mol) | rmsd l.b.| rmsd u.b.

-----+------------+----------+----------

1 -7.151 0 0

2 -7.041 3.445 4.637

3 -7.014 2.577 6.601

4 -6.944 3.089 6.603

5 -6.772 2.894 5.377

6 -6.702 2.966 6.743

7 -6.695 21.18 24.1

8 -6.642 1.759 2.444

9 -6.587 3.029 4.497
